# Black soldier fly larvae meal influences the growth, reproduction and related gene expression in farm-raised and growth-trait-selected *Clarias magur* brooders

**DOI:** 10.1038/s41598-025-30296-8

**Published:** 2025-12-20

**Authors:** Saiprasad Bhusare, Chandan G. M., Parimal Sardar, Narottam Prasad Sahu, Selvaraju Sellappan, Muralidhar P. Ande, Kedar Nath Mohanta, Tejaswini Kinnera, Nazeemashahul Shamna

**Affiliations:** 1 ICAR-Central Institute of Fisheries Education, Mumbai, Maharashtra India; 2 ICAR-National Institute of Animal Nutrition and Physiology, Bengaluru, India; 3 FWFF, Balabhadrapuram, Kakinada Centre, ICAR-Central Institute of Fisheries Education, Kakinada, India

**Keywords:** Black soldier fly larvae meal, Maha magur, Reproductive traits, Larval survival, Sex steroid hormone, Ichthyology, Reproductive biology

## Abstract

A 120-day feeding trial was conducted to evaluate the effects of Black Soldier Fly Larvae (BSFL) meal on gonadal development, reproductive performance, and gene expression in two populations of *Clarias magur*: Farm-raised stock (FR) and genetically selected stock (GS), Maha Magur. Two diets, FM (14.65% fishmeal) and IM (20% BSFL, 0% fishmeal), were employed in a 2 × 2 factorial design. A total of 168 brooders were fed respective diets on a satiation basis. GS showed significantly better growth and nutrient utilization (final weight, weight gain %, SGR, PER) than FR (*p* < 0.05). Conversely, FR showed superior reproductive indices (GSI, Relative fecundity), reproductive performances (fertilization %, hatching % and larval survival %) (*p* < 0.05). Hormonal profiles varied among stocks, with FR having higher serum 17α20βDHP and GS females having increased estradiol. Serum 11-Keto-testosterone was increased in FR females and GS males. Gene expression indicated upregulation of *fshr*,* cyp19a1a*, and *vtg* in GS females, which correlated with increased estradiol, while increased *lhr* expression, correlating with serum 17α20β-DHP found in FR females. Histology indicated mature oocytes and numerous spermatozoa in FR, with GS females having vitellogenic oocytes. BSFL addition had little effect on the reproductive characters of either stock. In general, FR exhibited better reproductive performance and maturity irrespective of GS exhibiting improved growth.

**Supplementary Information:**

The online version contains supplementary material available at 10.1038/s41598-025-30296-8.

## Introduction

With the mounting pressure for high-quality proteins, India’s aquaculture industry is poised to experience strong growth in the years ahead. To fulfil this rising demand, the industry must enhance the production and productivity of fish and shellfish from culture systems. In addition to maximizing production, ensuring sustainability of aquafarms requires reduction of disease outbreaks and maintenance of long-term viability as crucial aspects in modern day intensified aquaculture. There is an urgent need to expand the diversity of species cultivated, as Indian aquaculture continues to be heavily reliant on carp and shrimp. Catfish farming has become one of the top aquaculture sectors globally, ranking among the top 10 species cultivated after carp and tilapia^1^.

Catfish constitute more than 12% of the teleost population and belong to the order Siluriformes, which consists of over 4,000 species^2^. They hold significant commercial value and are widely distributed^3^. In India, *Clarias magur* ranks among the most important catfish species for aquaculture. It has good market demand, nutritional value, and therapeutic use^4^. Worldwide, *Clarias magur* is the third most commonly cultured species, next to carp and tilapia^5^. The farm-raised population of *C. magur* has decreased considerably, and it has been classified as “threatened” by the International Union for Conservation of Nature (IUCN) and categorized as endangered by the ICAR-National Bureau of Fish Genetic Resources (NBFGR)^6,7^. The Government of India has recently prioritized catfish culture, leading to sector diversification. Despite that, studies on the aquaculture potential of native catfish species in India are scarce^8^.

As there is a huge demand for magur in India, for getting faster growth and profit, farmers rely on African catfish or hybrids to meet the demand in a shorter culture period. Even though African catfish is banned in India, many farmers culture it due its quick growth^9^. Therefore, it became essential to find a solution to enhance the harvest body weight of indigenous magur and to conserve indigenous catfish (*C. magur*), the ICAR-Central Institute of Fisheries Education, Mumbai, India (ICAR-CIFE) has started a genetic selection program to select a fast-growing family of *C. magur* (Maha Magur)^10^. The genetically selected stock (GS) of *C. magur* used in this study has undergone an ongoing selection program and has demonstrated superior progeny inheritance, particularly in growth traits, compared to the farm-raised stock (FR)/non-selected stock, making it a suitable candidate for evaluating the impacts of various feed types on growth and reproductive performance. The lack of effective breeding technologies and the limited supply of natural seeds present another significant obstacle to the expansion of *C. magur* culture. Poor broodstock quality and decline in natural stocks exacerbate this problem^11^. Since the animals have been selected for faster growth, evaluating reproductive efficiency and other related parameters is critical to ensure the long-term viability of the selected strain. Hence, quality brooders of *C. magur* must be developed to promote commercial aquaculture. High larval mortality, especially during the transition from wild prey to an artificial diet, has compounded the seed deficit^12^. Improvement of seed production and genetic improvement of *C. magur* with respect to growth and reproduction will play a crucial role in expanding magur culture. In addition, enhancing non-genetic factors, such as the rearing environment and feed quality, will increase larval survival. High-quality, stage-specific feed improves seed production and enhances genetic selection programs. Hence, understanding the digestibility and nutrient utilization of feed is essential for maximizing growth and reproductive potential.

The macronutrient requirements of genetically selected *C. magur* (Maha Magur) were previously reported^13^. *C. magur* is an insectivorous species, particularly during the breeding season, exhibiting a clear preference for insect larvae^14^. The success of *C. magur* reproduction depends largely on insect availability, as it is naturally insectivorous, with insect meal being a key diet ingredient. Black soldier fly (BSF) larvae are highly sustainable due to their ability to convert organic waste into proteins, lipids, and chitin^15,16^. Black Soldier Fly (BSF) larvae culture is highly scalable and can be adapted for large-scale commercial production^17^. The use of waste as feed further reduces costs, making BSF an economically sustainable and scalable source of protein for aquaculture^18,19^. According to previously reported study the inclusion of *Hermetia illucens*, Black Soldier Fly Larvae (BSFL) at 21% enhanced reproductive efficiency in Maha Magur^13^. Thus, feeding both farm-raised and genetically selected stocks with a high-quality diet may show variations in reproductive efficiency.

Therefore, the current study aimed to evaluate the effect of fish meal-based and BSFL meal-based diets on somatic and gonadal growth, along with reproductive performance, in *Clarias magur* brooders from two distinct populations: the farm-raised stock (FR)/non-selected and the genetically selected stock (GS), known as Maha Magur. The term " farm-raised stock” refers to *Clarias magur* populations that have not undergone any genetic selection for growth traits, distinguishing them from the genetically selected Maha Magur stock.

## Results

### Proximate & fatty acid composition of feeds

**Fig. 1:**
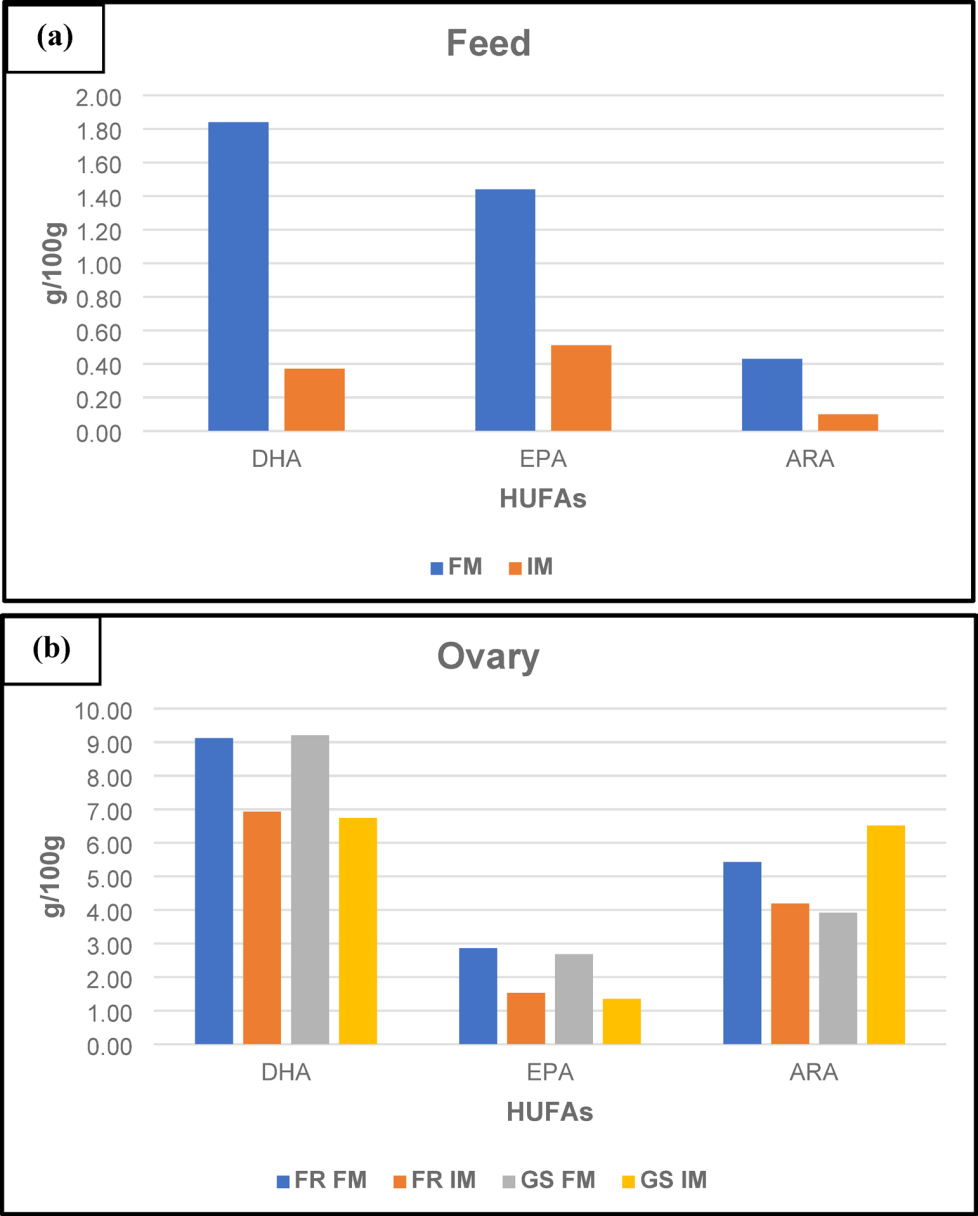
Fatty acid composition of experimental diets used and the ovaries of different *Clarias magur* broodstocks reared for a period of 120 days under the provision of feeding different experimental diets. FR FM, farm-raised stock (non-selected stock) fed with no BSFL meal; FR IM, farm-raised stock (non-selected stock) fed with 20% BSFL meal; GS FM, genetically improved stock fed with no BSFL meal; GS IM, genetically improved stock fed with 20% BSFL meal; ^2^FM, diet with no BSFL meal; IM, diet with 20% BSFL meal; DHA, Docosahexaenoic acid; EPA, Eicosapentanoic acid; ARA, Arachidonic acid.

The proximate analysis showed that the level of 36% crude protein and 8.5% ether extract was present in FM and IM based diet (Table 1). The replacement of FM with BSFL meal in the diet influenced the fatty acid profile of the diets (Table 2). In the diet specifically, the total saturated fatty acid (ΣSFA) content significantly increased (*p* < 0.05) in the insect meal (IM)-based diet with the BSFL meal. Within the ΣSFA fraction, lauric acid (C12) exhibited the most significant increase, followed by that of myristic acid. Conversely, total monounsaturated fatty acid (ΣMUFA) content significantly decreased (*p* < 0.05). There was a significant decrease (*p* < 0.05) in the amounts of polyunsaturated fatty acids like linoleic acid 18:2(n-6) and linolenic acid 18:3(n-3) in the insect meal-based diet. The amount of highly unsaturated fatty acids (HUFAs), including of EPA (eicosapentaenoic acid), DHA (docosahexaenoic acid) and ARA (arachidonic acid) were found to be lower (Fig. 1a).

**Table 1:** Formulation and proximate composition of experimental diets fed to *Clarias Magur* broodstock cultured for the period of 120 days.

Ingredients (%)	Diets (Treatments)^1^	
	FM	IM
Fish Meal^2^	14.65	0.00
BSFL Meal^3^	0.00	20.00
DSBM^4^	25.00	25.00
Groundnut Oil Cake^4^	25.00	25.00
De-Oiled Rice Bran^4^	14.48	10.73
Wheat Flour^5^	6.00	6.00
Corn Flour^5^	6.00	6.00
Fish Oil^2^	2.40	1.60
Veg Oil^5^	2.40	1.60
Vit-Min Mix^6^	2.00	2.00
CMC^7^	1.50	1.50
BHT^7^	0.02	0.02
Choline chloride^7^	0.20	0.20
Stay C^8^	0.30	0.30
Vit-E^8^	0.05	0.05
**Proximate Composition (% dry weight basis)**		
Dry matter	93.66	93.24
Crude protein (CP)	36.27	36.15
Ether extract	8.54	8.52
Crude fibre	5.92	7.15
Total ash	8.45	9.96
NFE^9^	40.82	38.23
DE^10^ (Kcal/100 g)	385.25	374.16

^1^FM, diet with no BSFL meal; IM, diet with 20% BSFL meal;.

^2^Fish meal and fish oil were purchased from T J Marine Industry, Ratnagiri, India (CP% of FM = 60.84%); ^3^BSFL Meal, Black Soldier Fly Larvae Meal (IM), was purchased from Kovai BSFL, Coimbatore, India (CP% of IM = 44.52%); ^4^DSBM, De-fatted soybean meal, GNOC, Groundnut oil cake, DORB, De-oiled rice bran and was purchased from a local market in Kakinada, India. ^5^Wheat flour, Corn flour and Vegetable oil were purchased from D-Mart, Mumbai, India; ^6^Composition of vitamin-mineral mix (AGRIMIN FORTE) (quantity kg^− 1^): Vitamin A, 7,00,000 IU; Vitamin D3, 70,000 IU; Vitamin E, 250 mg; Nicotinamide, 1000 mg; Cobalt, 150 mg; Copper, 1200 mg; Iodine, 325 mg; Iron, 1500 mg; Mg, 6000 mg; Mn, 1500 mg; K, 100 mg; Na, 5.9 mg; S, 0.72%; Zn, 9600 mg; Ca, 25.5%; P, 12.75%; ^7^CMC, Carboxymethyl cellulose, BHT, Butylated hydroxytoluene and Choline chloride were purchased from Himedia Laboratories, Mumbai, India; ^8^Stay C and Vit-E were purchased from SLR, Mumbai, India.

^9^NFE %, Nitrogen free extract, = 100 – (% Crude Protein + % Ether Extract + % Crude Fiber + % Ash); ^10^DE, digestible energy = Crude Protein (%) x 4 + Ether Extract (%) x 9 + Nitrogen Free Extract (%) x 4.

**Table 2:** Effects of experimental diets on fatty acid composition of ovaries of different *Clarias Magur* broodstocks reared for the period of 120 days.

Fatty acid composition (g/100 g)	Diet^2^			Treatments (Ovary)^1^				
	Diet FM	Diet IM	*p*-value	FR FM	FR IM	GS FM	GS IM	*p*-value
C-12:0 Lauric Acid	0.30^a^ ± 0.02	13.77^b^ ± 0.48	***< 0.001***	0.00	1.11^c^ ± 0.05	0.06^a^ ± 0.01	0.98^b^ ± 0.04	***< 0.001***
C-14:0 Myristic Acid	1.27^a^ ± 0.03	2.93^b^ ± 0.05	***< 0.001***	1.32^a^ ± 0.03	3.06^d^ ± 0.06	1.52^b^ ± 0.02	2.78^c^ ± 0.04	***< 0.001***
C-16:0 Palmitic Acid	13.69 ± 0.07	13.51 ± 0.06	***0.117***	21.81^c^ ± 0.07	20.78^ab^ ± 0.06	20.57^a^ ± 0.04	20.98^b^ ± 0.08	***< 0.001***
C-17:0 Heptadecanoic Acid	0.25^b^ ± 0.02	0.10^a^ ± 0.02	***0.005***	1.61^d^ ± 0.03	0.96^a^ ± 0.01	1.10^b^ ± 0.06	1.49^c^ ± 0.04	***< 0.001***
**C-20:0 Arachidic Acid**	0.58^b^ ± 0.02	0.39^a^ ± 0.03	***0.005***	0.08^c^ ± 0.01	0.00^a^ ± 0.00	0.06^b^ ± 0.01	0.00	***< 0.001***
C-22:0 Behenic Acid	0.30^b^ ± 0.02	0.19^a^ ± 0.01	***0.007***	0.00	0.00	0.00	0.00	***-***
**C-24:0 Lignoceric Acid**	0.18^b^ ± 0.02	0.08^a^ ± 0.01	***0.010***	BLQ	BLQ	BLQ	BLQ	***-***
C-16:1 Palmitoleic Acid	1.34^b^ ± 0.05	1.11^a^ ± 0.04	***0.026***	2.18^c^ ± 0.05	1.75^a^ ± 0.06	2.53^d^ ± 0.03	1.91^b^ ± 0.04	***< 0.001***
C-18:2 (n-6) - Linoleic acid	36.45^b^ ± 0.48	31.11^a^ ± 0.36	***0.001***	12.43^b^ ± 0.26	14.00^c^ ± 0.30	13.49^c^ ± 0.35	11.42^a^ ± 0.24	***0.001***
**C-18:3 (n-3) Linolenic Acid**	3.77^b^ ± 0.06	2.72^a^ ± 0.04	***< 0.001***	2.97^c^ ± 0.08	2.40^b^ ± 0.07	2.97^c^ ± 0.08	1.73^a^ ± 0.04	***< 0.001***
**C-20:2 (n-6) dihomolinoleic acid**	0.00	0.00	***-***	0.66^b^ ± 0.02	0.41^a^ ± 0.02	0.46^a^ ± 0.03	0.42^a^ ± 0.03	***< 0.001***
**C-20:3 (n-6)dihomo-γ-linolenic acid (DGLA)**	0.06^b^ ± 0.01	0.00^a^	***0.003***	2.09^b^ ± 0.04	3.34^c^ ± 0.04	1.89^a^ ± 0.02	3.68^d^ ± 0.03	***< 0.001***
**C-20:4 (n-6) Arachidonic Acid**	0.44^b^ ± 0.03	0.10^a^ ± 0.02	***< 0.001***	5.43^c^ ± 0.04	4.19^b^ ± 0.08	3.92^a^ ± 0.09	6.51^d^ ± 0.06	***< 0.001***
**C-20:5 (n-3) Eicosapentanoic (EPA)**	1.44^a^ ± 0.04	0.51^b^ ± 0.03	***< 0.001***	2.86^d^ ± 0.06	1.53^b^ ± 0.03	2.68^c^ ± 0.05	1.35^a^ ± 0.03	***< 0.001***
**C-22:1 (n-6) Eicosenoic Acid**	0.28^b^ ± 0.02	0.16^a^ ± 0.01	***0.005***	0.59^b^ ± 0.02	0.51^a^ ± 0.02	0.60^b^ ± 0.02	0.76^c^ ± 0.02	***< 0.001***
C-22:6 (n-3) Docosahexaenoic Acid (DHA)	1.84^b^ ± 0.04	0.37^a^ ± 0.03	***< 0.001***	9.12^c^ ± 0.05	6.93^b^ ± 0.06	9.20^c^ ± 0.02	6.74^a^ ± 0.06	***< 0.001***
Ʃ Saturated Fatty Acids (SFA)	23.23^a^ ± 0.17	36.05^b^ ± 0.17	***< 0.001***	37.46^b^ ± 0.24	38.92^c^ ± 0.17	36.21^a^ ± 0.14	38.56^c^ ± 0.17	***< 0.001***
Ʃ Unsaturated Fatty Acids (USFA)	76.75^b^ ± 0.20	63.93^a^ ± 0.40	***< 0.001***	60.34^b^ ± 0.15	59.05^a^ ± 0.36	61.99^c^ ± 0.14	58.62^a^ ± 0.37	***< 0.001***
Ʃ Monounsaturated Fatty Acids (MUFA)	32.76^b^ ± 0.21	29.13^a^ ± 0.06	***< 0.001***	25.43^a^ ± 0.30	26.66^b^ ± 0.18	27.84^c^ ± 0.16	27.19^bc^ ± 0.18	***< 0.001***
Ʃ Polyunsaturated Fatty Acids (PUFA)	44.27^b^ ± 0.38	34.96^a^ ± 0.34	***< 0.001***	36.16^b^ ± 0.44	33.32^a^ ± 0.20	35.21^b^ ± 0.27	32.61^a^ ± 0.19	***< 0.001***
Ʃ Omega 3 Fatty Acids PUFA	7.05^b^ ± 0.05	3.59^a^ ± 0.03	***< 0.001***	14.95^c^ ± 0.19	10.86^b^ ± 0.15	14.85^c^ ± 0.06	9.82^a^ ± 0.02	***< 0.001***
Ʃ Omega 6 Fatty Acids PUFA	36.88^b^ ± 0.45	31.21^a^ ± 0.35	***0.001***	17.87 ± 0.30	18.19 ± 0.38	17.41 ± 0.27	17.93 ± 0.18	***0.349***
Omega 3: Omega 6 (n3/n6)	0.19^b^ ± 0.01	0.11^a^ ± 0.00	***< 0.001***	0.84^c^ ± 0.00	0.60^b^ ± 0.02	0.85^c^ ± 0.02	0.55^a^ ± 0.01	***< 0.001***
Omega 6: Omega 3 (n6/n3)	5.24^a^ ± 0.10	8.69^b^ ± 0.12	***< 0.001***	1.20^a^ ± 0.00	1.68^b^ ± 0.06	1.17^a^ ± 0.02	1.82^c^ ± 0.02	***< 0.001***
ARA/EPA	0.30^b^ ± 0.03	0.19^a^ ± 0.02	***0.033***	-	-	-	-	***-***

Data are expressed as mean ± S.E, *n* = 3 (One-way ANOVA); mean values in the same column with different superscripts differ significantly (*p* < 0.05).

^1^FR FM, farm-raised stock (non-selected stock) fed with no BSFL meal; FR IM, farm-raised stock (non-selected stock) fed with 20% BSFL meal; GS FM, genetically improved stock fed with no BSFL meal; GS IM, genetically improved stock fed with 20% BSFL meal; ^2^FM, diet with no BSFL meal; IM, diet with 20% BSFL meal;.

BLQ, below limit of quantification; ƩSFA, total saturated fatty acid; ƩUSFA, total unsaturated fatty acid; ƩMUFA, total mono unsaturated fatty acid; ƩPUFA, total poly unsaturated fatty acid.

### Growth and nutrient utilization

The results of the two-way ANOVA suggest that genetic stock (GS) significantly affected final body weight (FBW), weight gain (WG), weight gain percentage (WG%), specific growth rate (SGR), feed conversion ratio (FCR) and protein efficiency ratio (PER) (Table 3). Conversely, replacement of fish meal (FM) with insect meal (IM) did not exhibit any statistically significant difference in FBW, WG, WG%, SGR, PER, or FCR. The magur broodstock from GS exhibited significantly higher (*p* < 0.05) FBW, WG, WG%, SGR, and PER, along with a lower FCR, compared to those from the farm-raised stock (FR). Additionally, no significant interaction effects were observed for any of the parameters. One-way ANOVA further confirmed that GS magur fed both FM and IM-based diets demonstrated significantly higher (*p* < 0.05) FBW, WG, WG%, SGR, and PER, as well as a lower FCR.

**Table 3:** Effects of experimental diets on growth and nutrient utilization of different *Clarias Magur* broodstocks reared for the period of 120 days.

Treatments^1^	IBW^2^ (g)	FBW^3^ (g)	WG^4^ (g)	% WG^5^	SGR^6^	FCR^7^	PER^8^	CF^9^
**One Way ANOVA**								
FR FM	134.33^a^ ± 1.34	174.31^a^ ± 3.31	39.98^a^ ± 3.84	29.79^a^ ± 3.06	0.22^a^ ± 0.02	2.90^b^ ± 0.21	0.97^a^ ± 0.07	0.95 ± 0.18
FR IM	131.19^a^ ± 1.30	169.41^a^ ± 4.25	38.21^a^ ± 3.37	29.11^a^ ± 2.41	0.21^a^ ± 0.02	3.01^b^ ± 0.26	0.92^a^ ± 0.09	1.18 ± 0.06
GS FM	198.33^b^ ± 1.11	271.71^b^ ± 4.32	73.38^b^ ± 3.33	36.98^b^ ± 1.50	0.26^b^ ± 0.01	2.26^a^ ± 0.12	1.23^b^ ± 0.07	1.32 ± 0.19
GS IM	200.64^b^ ± 2.03	271.64^b^ ± 2.43	71.00^b^ ± 3.65	35.42^b^ ± 2.11	0.25^b^ ± 0.01	2.35^a^ ± 0.20	1.18^b^ ± 0.10	0.97 ± 0.05
***p-value***	***< 0.001***	***< 0.001***	***< 0.001***	***0.014***	***0.022***	***0.019***	***0.017***	***0.253***
**Two Way ANOVA**								
**Effect of different magur stocks**								
Farm-raised stock	132.76^a^	169.69^a^	36.930^a^	27.82^a^	0.21^a^	2.96^a^	0.94^a^	1.13^a^
Genetic stock	199.49^b^	272.520^b^	73.040^b^	36.63^b^	0.26^b^	2.15^b^	1.30^b^	1.47^b^
*SEM*	*1.049*	*2.276*	*2.150*	*1.403*	0.009	*0.137*	*0.059*	0.052
***p-value***	***< 0.001***	***< 0.001***	***< 0.001***	***0.002***	***0.003***	***0.003***	***0.003***	***0.002***
**Effect of different feeds**								
Fish-meal	166.33	222.21	55.88	32.71	0.24	2.51	1.15	1.28
Insect-meal	165.92	220.00	54.09	31.73	0.23	2.60	1.09	1.32
*SEM*	*1.049*	*2.276*	*2.150*	*1.403*	*0.009*	*0.137*	*0.059*	*0.052*
***p-value***	***0.785***	***0.511***	***0.571***	***0.633***	***0.717***	***0.671***	***0.530***	***0.598***
**Interaction effect of different** **magur stocks X feeds**								
***p-value***	***0.104***	***0.459***	***0.943***	***0.841***	***0.904***	***0.913***	***0.954***	***0.859***

Data are expressed as mean ± SE, *n* = 3 (One-way ANOVA) and mean & SEM, *n* = 3 (Two-way ANOVA); mean values in the same column with different superscripts differ significantly (*p* < 0.05).

^1^FR FM, farm-raised stock (non-selected stock) fed with no BSFL meal; FR IM, farm-raised stock (non-selected stock) fed with 20% BSFL meal; GS FM, genetically improved stock fed with no BSFL meal; GS IM, genetically improved stock fed with 20% BSFL meal; ^2^FM, diet with no BSFL meal; IM, diet with 20% BSFL meal;.

^2^IBW, Initial mean body weight; ^3^FBW, Final mean body weight; ^4^WG, weight gain; ^5^%WG, weight gain percentage; ^6^SGR, specific growth rate, % per day; ^7^FCR, feed conversion ratio; ^8^PER, protein efficiency ratio; ^9^CF, Condition factor.

### Reproductive and somatic indices

The female magur broodstock from the GS exhibited significantly higher (*p* < 0.05) HSI (hepatosomatic index) and absolute fecundity, whereas the female broodstock from the farm-raised stock (FR) demonstrated significantly higher (*p* < 0.05) GSI (gonadosomatic index) and relative fecundity (Table 4). In males, GSI was significantly higher (*p* < 0.05) in GS, while HSI did not differ significantly across stocks and treatments. The replacement of fish meal (FM) with insect meal (IM) did not result in any statistically significant differences in the HSI, GSI, absolute fecundity, or relative fecundity. One-way ANOVA further confirmed that GSI and relative fecundity were significantly higher (*p* < 0.05) in female Magur broodstock from FR than in GS. HSI and absolute fecundity were both greater (*p* < 0.05) in GS females, irrespective of diet. The IM group within the GS had a significantly higher GSI in males (*p* < 0.05). However, there were no major differences seen in the HSI.

**Table 4:** Effects of experimental diets on reproductive and somatic indices of different *Clarias Magur* broodstocks reared for the period of 120 days.

	Female ♀				Male ♂	
**Treatments** ^**1**^	**HSI** ^**2**^	**GSI** ^**3**^	**Absolute fecundity** ^**4**^	**Relative fecundity** ^**5**^	**HSI** ^**2**^	**GSI** ^**3**^
**One Way ANOVA**						
FR FM	0.65^a^ ± 0.02	11.79^c^ ± 0.88	6316^a^ ± 261	4675^b^ ± 125	0.60 ± 0.02	0.42^ab^ ± 0.02
FR IM	0.67^ab^ ± 0.01	11.00^bc^ ± 0.67	6160^a^ ± 251	4415^b^ ± 252	0.62 ± 0.01	0.39^a^ ± 0.03
GS FM	0.72^bc^ ± 0.03	8.31^a^ ± 0.56	7842^b^ ± 255	3333^a^ ± 137	0.65 ± 0.03	0.49^bc^ ± 0.02
GS IM	0.75^c^ ± 0.02	8.92^ab^ ± 0.43	8243^b^ ± 296	3506^a^ ± 119	0.69 ± 0.02	0.51^c^ ± 0.03
***p-value***	***0.018***	***0.016***	***0.001***	***0.001***	***0.090***	***0.016***
**Two Way ANOVA**						
**Effect of different magur stocks**						
Farm-raised stock	0.66^a^	11.54^b^	7847^b^	4611^b^	0.62	0.40^a^
Genetic stock	0.73^b^	8.67^a^	9242^a^	3446^a^	0.67	0.49^b^
*SEM*	*0.012*	*0.384*	*215*	*119*	*0.015*	*0.014*
***p-value***	***0.004***	***0.001***	***0.002***	***< 0.001***	***0.051***	***0.003***
**Effect of different feeds**						
Fish-meal	0.68	10.13	8488	4046	0.63	0.45
Insect-meal	0.70	10.07	8600	4011	0.66	0.44
*SEM*	*0.012*	*0.384*	*215*	*119*	*0.015*	*0.014*
***p-value***	***0.226***	***0.912***	***0.722***	***0.840***	***0.258***	***0.936***
**Interaction effect of different magur stocks X feeds**						
***p-value***	***0.627***	***0.305***	***0.416***	***0.327***	***0.696***	***0.315***

Data are expressed as mean ± SE, *n* = 3 (One-way ANOVA) and mean & SEM, *n* = 3 (Two-way ANOVA); mean values in the same column with different superscripts differ significantly (*p* < 0.05).

^1^FR FM, farm-raised stock (non-selected stock) fed with no BSFL meal; FR IM, farm-raised stock (non-selected stock) fed with 20% BSFL meal; GS FM, genetically improved stock fed with no BSFL meal; GS IM, genetically improved stock fed with 20% BSFL meal; ^2^FM, diet with no BSFL meal; IM, diet with 20% BSFL meal;.

^2^HSI, Hepatosomatic index; ^3^GSI, Gonadosomatic index; ^4^AF, Absolute fecundity (number of eggs per total weight of fish); ^5^RF, Relative fecundity (number of eggs per 100 g body weight of fish).

### Fatty acid composition of ovary

The concentrations of 18:2(n-6) linoleic acids were highest in the FRIM group and 18:3(n-3) linolenic acid was increased in both stocks fed FM-based diets (FRFM and GSFM) (Table 3). Similarly, the levels of 20:5(n-3) eicosapentaenoic acid (EPA) and 22:6(n-3) docosahexaenoic acid were increased in both stocks fed FM-based diets (FRFM and GSFM). However, ARA levels were higher in the genetic stocks fed with IM-based diets (GSIM group). Besides, considering the DHA: EPA: ARA, irrespective of dietary treatments and the stocks, DHA levels were increased and conserved in the ovaries, maintaining similar values across all treatments and the stocks. In contrast, EPA concentrations were lower in ovarian tissue compared to dietary supplementation, while ARA levels were higher than those observed in the respective diets as shown in the Fig. 1b. Synthesis and conservation of ARA in the ovary is evident by the presence of new fatty acids, especially the n-6 series, such as C-20:2 (n-6) dihomolinoleic acid, C-20:3 (n-6), and C-20:3 (n-6) dihomo-γ-linolenic acid (DGLA) were observed in ovarian fatty acid analysis.

The total n-3 polyunsaturated fatty acids (∑n-3 PUFA) were found to be increased in FM-based diet fed treatments in both the stocks, while there was no change in total n-6 polyunsaturated fatty acids (∑n-6) across the stocks and treatments. Consequently, the n-3/n-6 ratio was lowest in the IM-based diet-fed group in either of the stocks, whereas the n-6/n-3 ratio was highest in this group fed with IM-based diets.

### Reproductive performance

The results of the two-way ANOVA revealed that the stock had a significant effect (*p* < 0.05) on reproductive parameters, including the ovi-somatic index (OSI), fertilization rate (%), hatching rate (%), and larval survival (%) (Table 5). However, replacing FM protein with IM did not significantly influence any of these parameters. Magur broodstock from the FR stock exhibited significantly higher OSI, fertilization rate, hatching rate, and larval survival (*p* < 0.05) than the GS stock. In contrast, egg weight and egg diameter did not vary significantly between the two stocks. No significant interaction effects were detected between the stock type and diet (FM and IM-based diets) for any of the analyzed reproductive parameters. Further analysis using one-way ANOVA confirmed that OSI, fertilization rate, hatching rate, and larval survival were significantly higher (*p* < 0.05) in magur broodstock from the FR stock than in the GS stock, regardless of the dietary treatment. However, egg weight and egg diameter remained statistically non-significant across both stock types and dietary groups.

**Table 5:** Effects of experimental diets on reproductive performance of different *Clarias Magur* broodstocks reared for the period of 120 days.

Treatments^1^	OSI^2^ (%)	Egg weight (mg)	Egg size (mm)	Fertilisation rate (%)	Hatching rate (%)	3rd day larval survival (%)	7th day larval survival (%)
**One Way ANOVA**							
FR FM	11.50^c^ ± 0.94	2.76 ± 0.15	1.73 ± 0.03	93.40^c^ ± 3.73	81.73^b^ ± 1.04	84.94^b^ ± 2.58	72.60^b^ ± 2.32
FR IM	10.87^bc^ ± 0.68	2.81 ± 0.19	1.82 ± 0.01	90.31^bc^ ± 6.09	77.56^b^ ± 2.28	79.41^b^ ± 1.65	69.74^b^ ± 5.08
GS FM	8.13^a^ ± 0.48	2.52 ± 0.17	1.79 ± 0.03	75.48^ab^ ± 4.42	63.75^a^ ± 2.12	62.97^a^ ± 4.68	53.57^a^ ± 1.10
GS IM	8.70^ab^ ± 0.50	2.56 ± 0.11	1.72 ± 0.03	70.36^a^ ± 4.07	66.54^a^ ± 1.40	63.63^a^ ± 2.33	51.34^a^ ± 3.67
***p-value***	**0.020**	**0.529**	**0.060**	**0.021**	**< 0.001**	**0.002**	**0.004**
**Two Way ANOVA**							
**Effect of different magur stocks**							
Farm-raised stock	11.19^b^	2.78	1.78	91.86^b^	79.65^b^	82.17^b^	71.17^b^
Genetic stock	8.41^a^	2.54	1.76	72.92^a^	65.15^a^	63.30^a^	52.46^a^
*SEM*	*0.400*	*0.112*	*0.018*	*3.300*	1.263	*2.142*	*2.395*
***p-value***	***0.001***	***0.167***	***0.490***	***0.004***	***< 0.001***	***< 0.001***	***< 0.001***
**Effect of different feeds**							
Fish-meal	9.82	2.64	1.76	84.44	72.74	73.96	63.09
Insect-meal	9.78	2.69	1.77	80.33	72.05	71.52	60.54
*SEM*	*0.400*	*0.112*	*0.018*	*3.300*	*1.263*	*2.142*	*2.395*
***p-value***	***0.948***	***0.775***	***0.570***	***0.404***	***0.707***	***0.445***	***0.473***
**Interaction effect of different magur stocks X feeds**							
***p-value***	***0.324***	***0.967***	***0.012***	***0.833***	***0.087***	***0.337***	***0.928***

Data are expressed as mean ± SE, *n* = 3 (One-way ANOVA) and mean & SEM, *n* = 3 (Two-way ANOVA); mean values in the same column with different superscripts differ significantly (*p* < 0.05).

^1^FR FM, farm-raised stock (non-selected stock) fed with no BSFL meal; FR IM, farm-raised stock (non-selected stock) fed with 20% BSFL meal; GS FM, genetically improved stock fed with no BSFL meal; GS IM, genetically improved stock fed with 20% BSFL meal; ^2^FM, diet with no BSFL meal; IM, diet with 20% BSFL meal;.

^2^OSI, Ovisomatic index.

### Serum sex steroid hormone

According to the two-way ANOVA analysis, serum sex steroid hormones, including estradiol, 17α20β-DHP, and 11-Keto-testosterones (11KT), displayed a significant (*p* < 0.05) variation in different stocks (Table 6). The estradiol level was significantly higher in the females with GS. Conversely, 17α20β-DHP and 11KT were significantly higher in females belonging to the FR group. In case of males, 17α20β-DHP was significantly (*p* < 0.05) higher in FR, but 11KT was higher in GS. Moreover, replacement of fish meal (FM) with insect meal (IM) did not result in any statistically significant differences in these serum sex steroid hormones in both females and males. Moreover, no significant interaction effects were observed between stock type and diet (FM and IM-based diets) for estradiol, or 17α20β-DHP, and 11KT, both in females and males. According to one-way ANOVA analysis, highest estradiol level was recorded in GS, and the highest 17α20β-DHP and 11KT levels were recorded in FR fed either of the diets (FM and IM-based diets). Additionally, in males, 17α20β-DHP was higher in FR, and 11 KT was higher in GS.

**Table 6:** Effects of experimental diets on sex steroid hormone profile of different *Clarias Magur* broodstocks reared for the period of 120 days.

	Female ♀			Male ♂	
**Treatments** ^**1**^	**Estradiol (pg/ml)**	**17α20β-DHP**^**2**^ **(mU/ml)**	**11-KT** ^**3**^ **(pg/ml)**	**17α20β-DHP**^**2**^ **(mU/ml)**	**11-KT**^**3**^ **(pg/ml)**
**One Way ANOVA**					
FR FM	307.19^a^ ± 12.63	267.10^b^ ± 16.24	62.03^b^ ± 1.15	294.88^b^ ± 16.24	45.95^a^ ± 1.49
FR IM	335.73^a^ ± 15.37	241.82^b^ ± 9.84	59.72^b^ ± 1.68	281.55^b^ ± 12.01	48.06^a^ ± 1.36
GS FM	550.94^b^ ± 15.99	194.97^a^ ± 10.21	48.08^a^ ± 2.22	223.67^a^ ± 9.38	56.45^b^ ± 1.68
GS IM	586.56^b^ ± 11.73	181.45^a^ ± 13.90	44.10^a^ ± 1.77	209.23^a^ ± 13.90	57.67^b^ ± 2.21
***p-value***	**< 0.001**	**0.005**	**< 0.001**	**0.004**	**0.003**
**Two Way ANOVA**					
**Effect of different magur stocks**					
Farm-raised stock	324.58^a^	254.46^b^	60.87^b^	288.21^b^	47.01^a^
Genetic stock	562.92^b^	188.21^a^	46.09^a^	216.45^a^	57.06^b^
*SEM*	*9.847*	*9.069*	*1.236*	*9.282*	1.214
***p-value***	***< 0.001***	***0.001***	***< 0.001***	***0.001***	***< 0.001***
**Effect of different feeds**					
Fish-meal	426.04	231.04	55.06	259.28	51.20
Insect-meal	461.46	211.64	51.91	245.39	52.87
*SEM*	*9.847*	*9.069*	*1.236*	*9.282*	*1.214*
***p-value***	***0.052***	***0.169***	***0.109***	***0.321***	***0.360***
**Interaction effect of different magur stocks X feeds**					
***p-value***	***0.807***	***0.659***	***0.644***	***0.967***	***0.801***

Data are expressed as mean ± SE, *n* = 3 (One-way ANOVA) and mean & SEM, *n* = 3 (Two-way ANOVA); mean values in the same column with different superscripts differ significantly (*p* < 0.05).

^1^FR FM, farm-raised stock (non-selected stock) fed with no BSFL meal; FR IM, farm-raised stock (non-selected stock) fed with 20% BSFL meal; GS FM, genetically improved stock fed with no BSFL meal; GS IM, genetically improved stock fed with 20% BSFL meal; ^2^FM, diet with no BSFL meal; IM, diet with 20% BSFL meal;.

^2^17α20β-DHP, 17α20β dihydroxyprogesterone; ^3^11-KT, 11 ketotestosterone.

### Serum biochemical parameters

Serum biochemical parameters varied significantly across the different stocks for both female and male broodstock Magurs according to the two-way ANOVA (Table 7). Specifically, serum glucose and TAG levels were significantly elevated in female magur broodstock of GS compared to the FR stock, whereas total cholesterol levels were significantly higher in female broodstock compared to the GS stock. In males, serum glucose and TAG levels did not exhibit significant variations, but total cholesterol levels were significantly higher in the GS stock. Results from the one-way ANOVA further confirmed that serum glucose and TAG levels were significantly higher (*p* < 0.05) in female magur broodstock than in the FR stock, while total cholesterol was highest in the GS stock. In contrast, serum glucose and TAG levels in males did not vary significantly; however, total cholesterol remained the highest in males from the GS stock, irrespective of the dietary treatment (FM or IM-based diets).

**Table 7:** Effects of experimental diets on serum glucose and lipid profile of different *Clarias Magur* broodstocks reared for the period of 120 days.

	Female ♀			Male ♂		
**Treatments** ^**1**^	**Glucose (mg/dl)**	**TAG**^**2**^ **(mg/dl)**	**Cholesterol (mg/dl)**	**Glucose (mg/dl)**	**TAG**^**2**^ **(mg/dl)**	**Cholesterol (mg/dl)**
**One Way ANOVA**						
FR FM	132.59^c^ ± 5.32	131.61^c^ ± 5.70	201.03^a^ ± 6.25	134.71 ± 3.59	139.80 ± 6.60	189.45^a^ ± 6.62
FR IM	126.03^bc^ ± 4.26	123.71^bc^ ± 4.07	209.78^ab^ ± 7.14	129.21 ± 4.26	129.60 ± 3.86	199.49^a^ ± 5.07
GS FM	106.14^a^ ± 2.90	104.31^a^ ± 5.46	232.17^c^ ± 4.93	125.93 ± 4.04	119.83 ± 2.49	222.40^b^ ± 7.42
GS IM	114.81^ab^ ± 5.01	111.35^ab^ ± 6.49	223.68^bc^ ± 6.26	123.28 ± 2.21	126.01 ± 4.21	211.33^ab^ ± 6.71
***p-value***	**0.013**	**0.031**	**0.030**	**0.215**	**0.074**	**0.033**
**Two Way ANOVA**						
**Effect of different magur stocks**						
Farm-raised stock	129.31^b^	127.66^b^	205.40^a^	131.96	134.70	194.47^a^
Genetic stock	110.48^a^	107.83^a^	227.93^b^	124.60	122.92	216.86^b^
*SEM*	*3.162*	*3.889*	*4.380*	*2.555*	3.208	*4.604*
***p-value***	***0.003***	***0.007***	***0.007***	***0.076***	***0.032***	***0.009***
**Effect of different feeds**						
Fish-meal	119.37	117.96	216.60	130.32	129.81	205.92
Insect-meal	120.42	117.53	216.73	126.24	127.80	205.41
*SEM*	*3.162*	*3.889*	*4.380*	*2.555*	*3.208*	*4.604*
***p-value***	***0.819***	***0.939***	***0.984***	***0.292***	***0.669***	***0.939***
**Interaction effect of different magur stocks X feeds**						
***p-value***	***0.127***	***0.211***	***0.201***	***0.703***	***0.109***	***0.144***

Data are expressed as mean ± SE, *n* = 3 (One-way ANOVA) and mean & SEM, *n* = 3 (Two-way ANOVA); mean values in the same column with different superscripts differ significantly (*p* < 0.05).

^1^FR FM, farm-raised stock (non-selected stock) fed with no BSFL meal; FR IM, farm-raised stock (non-selected stock) fed with 20% BSFL meal; GS FM, genetically improved stock fed with no BSFL meal; GS IM, genetically improved stock fed with 20% BSFL meal; ^2^FM, diet with no BSFL meal; IM, diet with 20% BSFL meal;.

^2^TAG, Serum triacylglycerol.

### mRNA expression of reproduction related genes

The results from the current study clearly demonstrate that the expression levels of genes involved in reproduction (Table 8), follicle-stimulating hormone receptor *(fshr*), luteinizing hormone receptor (*lhr*), cytochrome P450 aromatase (*cyp19a1a*), vitellogenin (*vtg*), and 11β-hydroxysteroid dehydrogenase (*11β-hsd*). In this study, FRFM (farm-raised stock fed with FM-based diet) was designated as the control group for comparison with the treatment groups and stocks. Consequently, the CT values of the control group were normalized to the CT values of the treatment groups. According to the two-way ANOVA analysis, in the female broodstock from the GS stock, the expression of *fshr*, *cyp19a1a*, and *vtg* was significantly higher (*p* < 0.05), whereas *lhr* expression was lower. Conversely, the FR stock exhibited an opposite expression pattern. Similarly, in the male broodstock, *fshr*, *cyp19a1a*, and *11β-hsd* expression levels were significantly higher (*p* < 0.05) in GS, while *lhr* expression was downregulated. The FR stock exhibited a reversed expression pattern compared to the GS stock. The replacement of fish meal (FM) protein with insect meal (IM) did not result in any statistically significant differences in the expression of most genes, except for *vtg* in females and *11β-hsd* in males, where the IM-based diet group exhibited significantly higher expression levels. There is no significant interaction effects were observed between stock type and diet (FM and IM-based diets) for any of the analyzed gene expression. Results from the one-way ANOVA further confirmed that in females, *fshr*, *cyp19a1a*, and *vtg* were significantly upregulated in the GS stock, while *lhr* was significantly upregulated in the FR stock. In males, *fshr*, *cyp19a1a*, and *11β-hsd* expression levels were significantly higher (*p* < 0.05) in the GS, whereas *lhr* expression was higher in the FR.

**Table 8:** Effects of experimental diets on relative mRNA expressions of genes involved in the steroidogenesis (11β HSD) and sex-hormones receptor (FSHR and LHR) of different *Clarias Magur* broodstocks reared for the period of 120 days.

	Female ♀				Male ♂			
**Treatments** ^**1**^	***fshr*** ^**2**^	***cyp19a1a*** ^**3**^	***lhr*** ^**4**^	***vtg*** ^**5**^	***fshr*** ^**2**^	***cyp19a1a*** ^**3**^	***lhr*** ^**4**^	***11β-hsd*** ^**6**^
**One Way ANOVA**								
FR FM	1.00^a^ ± 0.00	1.00^a^ ± 0.00	1.00^b^ ± 0.00	1.00^a^ ± 0.00	1.00 ± 0.00	1.00 ± 0.00	1.00^b^ ± 0.00	1.00^a^ ± 0.00
FR IM	1.12^a^ ± 0.08	1.17^a^ ± 0.08	0.93^b^ ± 0.05	1.43^b^ ± 0.11	1.10 ± 0.09	1.14 ± 0.08	0.93^b^ ± 0.05	1.21^b^ ± 0.06
GS FM	1.92^b^ ± 0.04	1.51^b^ ± 0.11	0.72^a^ ± 0.04	1.96^c^ ± 0.14	1.23 ± 0.06	1.27 ± 0.10	0.80^a^ ± 0.03	1.52^c^ ± 0.08
GS IM	2.03^b^ ± 0.06	1.70^b^ ± 0.08	0.63^a^ ± 0.03	2.33^c^ ± 0.15	1.31 ± 0.11	1.36 ± 0.09	0.72^a^ ± 0.04	1.68^c^ ± 0.05
***p-value***	**< 0.001**	**0.001**	**< 0.001**	**< 0.001**	**0.073**	**0.056**	**0.002**	**< 0.001**
**Two Way ANOVA**								
**Effect of different magur stocks**								
Farm-raised stock	1.06^a^	1.09^a^	0.96^b^	1.21^a^	1.05^a^	1.07^a^	0.96^b^	1.11^a^
Genetic stock	1.97^b^	1.61^b^	0.68^a^	2.14^b^	1.27^b^	1.31^b^	0.76^a^	1.60^b^
*SEM*	*0.038*	*0.056*	*0.025*	*0.084*	0.053	*0.056*	*0.026*	*0.038*
***p-value***	***< 0.001***	***< 0.001***	***< 0.001***	***< 0.001***	***0.018***	***0.016***	***0.001***	***< 0.001***
**Effect of different feeds**								
Fish-meal	1.46	1.26	0.86	1.48^a^	1.12	1.14	0.90	1.26^a^
Insect-meal	1.58	1.43	0.78	1.88^b^	1.21	1.25	0.82	1.45^b^
*SEM*	*0.038*	*0.056*	*0.025*	*0.084*	*0.053*	*0.056*	*0.026*	*0.038*
***p-value***	***0.054***	***0.055***	***0.063***	***0.011***	***0.260***	***0.188***	***0.068***	***0.008***
**Interaction effect of different magur stocks X feeds**								
***p-value***	***0.992***	***0.937***	***0.868***	***0.797***	***0.887***	***0.736***	***0.939***	***0.693***

Data are expressed as mean ± SE, *n* = 3 (One-way ANOVA) and mean & SEM, *n* = 3 (Two-way ANOVA); mean values in the same column with different superscripts differ significantly (*p* < 0.05).

^1^FR FM, farm-raised stock (non-selected stock) fed with no BSFL meal; FR IM, farm-raised stock (non-selected stock) fed with 20% BSFL meal; GS FM, genetically improved stock fed with no BSFL meal; GS IM, genetically improved stock fed with 20% BSFL meal; ^2^FM, diet with no BSFL meal; IM, diet with 20% BSFL meal;.

^2^*fshr*, Follicle stimulating hormone receptor; ^3^*cyp19a1a*, Cytochrome p450 aromatase; ^4^*lhr*, Luteinising hormone receptor; ^5^*vtg*, Vitellogenin; ^6^*11β-HSD*, 11β Hydroxysteroid dehydrogenase.

### Histological study of gonads

**Fig. 2:**
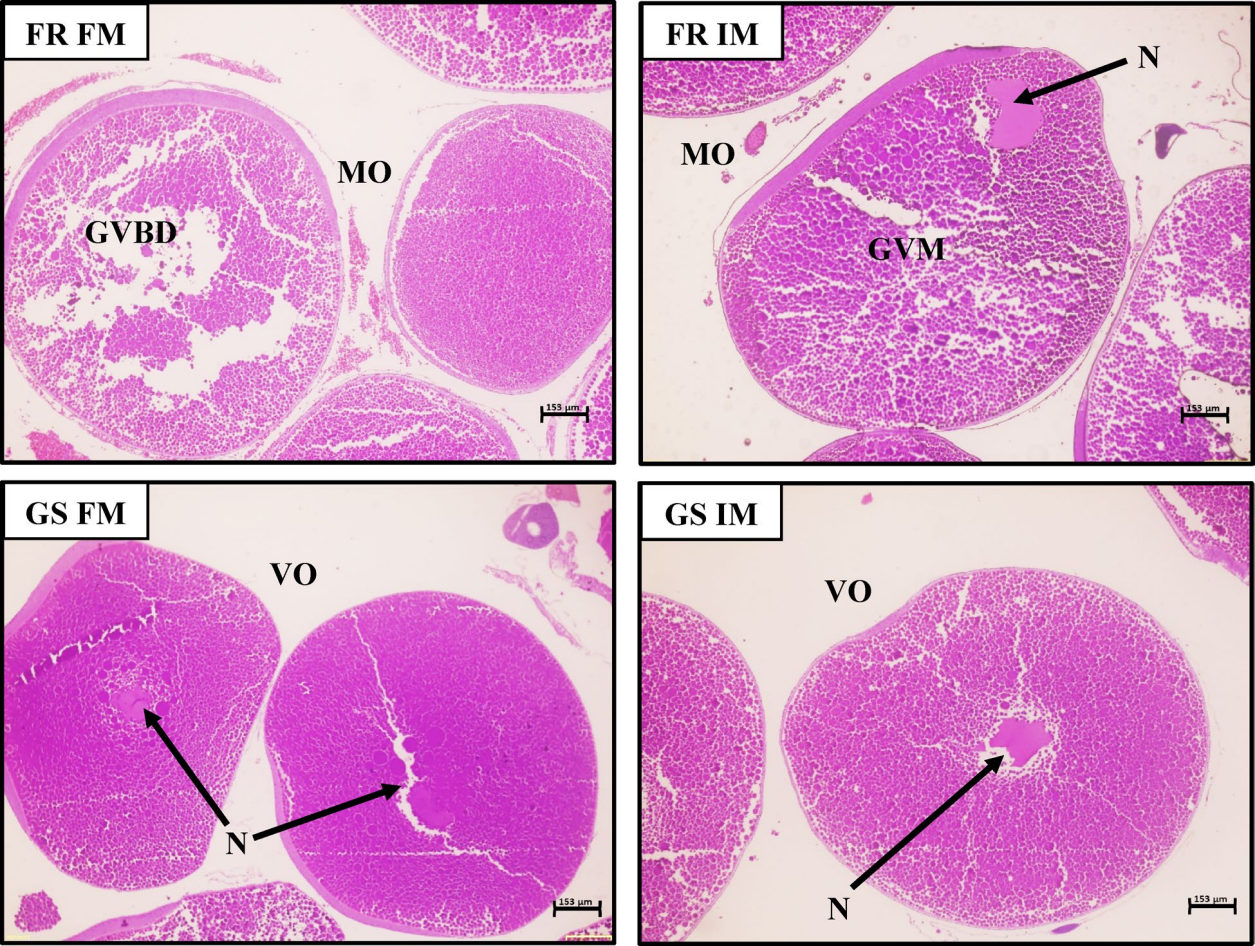
Histology of oocytes of different *Clarias magur* broodstocks fed different experimental diets for a period of 120 days. FR FM, farm-raised stock (non-selected stock) fed with no BSFL meal; FR IM, farm-raised stock (non-selected stock) fed with 20% BSFL meal; GS FM, genetically improved stock fed with no BSFL meal; GS IM, genetically improved stock fed with 20% BSFL meal; ^2^FM, diet with no BSFL meal; IM, diet with 20% BSFL meal; N = Nucleus; VO = Vitellogenic oocytes; MO = Mature oocytes in (GVM) germinal vesicle migration stage (GVBD) germinal vesicle breakdown stage and oocytes.

**Fig. 3:**
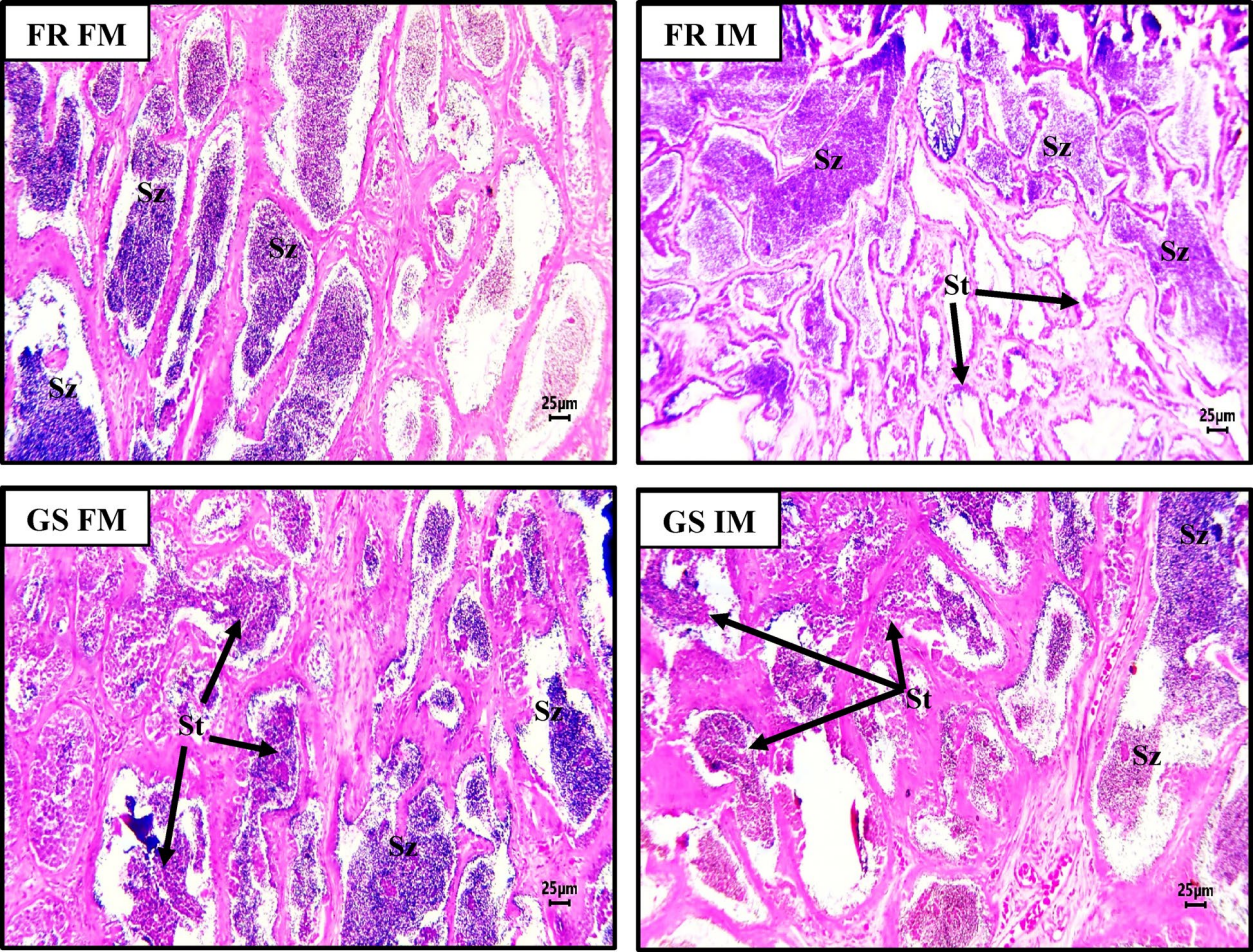
Histology of testis of different *Clarias magur* broodstocks fed different experimental diets for a period of 120 days. FR FM, farm-raised stock (non-selected stock) fed with no BSFL meal; FR IM, farm-raised stock (non-selected stock) fed with 20% BSFL meal; GS FM, genetically improved stock fed with no BSFL meal; GS IM, genetically improved stock fed with 20% BSFL meal; ^2^FM, diet with no BSFL meal; IM, diet with 20% BSFL meal; St = spermatids; Sz = spermatozoa.

The histology of the male and female gonads from magur broodstock fed FM and IM diets from both FR and GS stocks is shown in Figs. 2 and 3. Ovarian histology revealed different stages of oocyte development across stocks (Fig. 2).

In fish from the FR group, regardless of their diet, oocytes were found in the mature stage, with yolk globules and oil droplets in their cytoplasm. The FRFM group had mature oocytes at the germinal vesicle breakdown (GVBD) stage, while the FRIM group showed mature oocytes at the (germinal vesicle movement) GVM stage, with the nucleolus moving towards the animal pole. In contrast, fish from the GS stock (GSFM and GSIM) exhibited oocytes at the vitellogenic stage, where the nucleolus remained centrally positioned within the oocyte. Histological examination of testicular tissue revealed different stages of spermatogenesis in the male magur broodstock across the stocks (Fig. 3). In FR fish fed either FM or IM-based diets, the lumen of the seminiferous tubules contained dense spermatozoa, followed by the spermatids. However, in GS fish, the seminiferous tubule lumen contains a greater number of spermatids, followed by spermatozoa.

## Discussion

Selective breeding of fish is an effective strategy for developing strains with increased production capacity^20^, leading to improved strains like Maha Magur, which show greater growth rates in *Clarias magur*. However, factors such as environmental conditions, management, water quality, and diet influence fish’s ability to reach their genetic potential. Providing optimal culture conditions is vital for genetic improvement, as growth selection can impact reproductive efficiency and gamete quality^21^. This study aimed to assess the reproductive performance of farm-raised and genetically selected stocks of *C. magur* in response to two diet types.

In this study, genetically selected stocks showed significant increases in FBW, WG, WG%, SGR, and PER, along with reduced FCR, regardless of being fed fishmeal or insect meal-based diets. In contrast, farm-raised Magur stocks showed no significant dietary effect, with consistently lower growth and nutrient utilization, irrespective of the diets. This suggests that GS performed better than FR on both diets, indicating similar effects of both diets. Genetic variation in GS, selected for growth traits, improved performance^10^, similar to broiler studies^22,23^. Selective breeding has led to gains in production traits like fish fillet weight in tilapia, carp, and prawns without compromising quality^24^, as well as also noted improved feed efficiency in selectively bred fish^25^. One researcher observed high feed intake and efficiency in genetically selected tilapia strains^26^. This is preliminary study shows no difference between fishmeal and insect-based diets, suggesting 100% replacement of fishmeal protein with BSFL meal in Maha Magur is viable without affecting growth. Similarly, previous study reported that 75% fishmeal replacement with BSFL meal produced the highest WG and SGR in Nile tilapia fry^27^.

Polyunsaturated fatty acids (PUFAs) are crucial for fish reproduction, affecting gamete quality, gonadal development, spawning success, and embryo hatchability^28–31^. Neutral lipids, stored as oil droplets, increase in number as oocytes grow^32^. In this study, replacing fish meal with BSFL meal reduced dietary PUFAs, including LA (C18:2n-6), LNA (C18:3n-3), EPA (20:5n-3), DHA (C22:6n-3), and total n-3 and n-6 PUFAs, while increasing saturated fatty acids (SFAs) in both feed and ovaries. BSFL meal inclusion also decreased dietary arachidonic acid (ARA) (C20:4n-6) but increased ovarian ARA levels across treatment groups in both stocks (FR and GS). Higher ARA levels were found in the FR group fed FM-based diets and GS with IM-based diets, as most freshwater fish can biosynthesize ARA from LA and convert LNA to EPA and DHA^33,34^.

DHA, EPA, and ARA play significant roles in egg quality, hatching success, and larval viability^35,36^. Both the concentrations and ratios of the essential HUFAs (DHA, EPA, and ARA) influence fertilization rates and survival, suggesting that a high ARA: EPA in fish eggs may be mandatory for survival^37^. In the present study, an increase in ARA was observed in GS with a BSFL meal-based diet; however, FR showed the opposite trend. One researcher reported that the active production of eicosanoids from ARA and their involvement in reproductive processes^36^. Hence, in our study, DHA was conserved in the ovary for embryonic, larval, and neural development, and ARA played a role in prostaglandin synthesis, egg viability, and larval survival^37^. ARA plays a crucial role as a precursor to eicosanoids such as prostaglandins, thromboxanes, and leukotrienes involved in reproduction, egg development, and immunity^36,38,39^. Prostaglandins are essential for ovulation, female sexual behavior, and gonadotropin secretion^40^. The ARA-to-EPA ratio is significant in certain freshwater fish species due to ARA’s role in reproduction via prostaglandin synthesis^41^. Freshwater fish consume diets rich in n-6 fatty acids^33,34^. In this study, an insect-based diet had the highest n6/n3 ratio, influencing increased ovary n6/n3 ratio compared to fishmeal-based diets irrespective of stocks. Demersal freshwater spawners lack oil globules, so the accumulation of MUFAs in this species is not prominent^42^, explaining why MUFAs did not show differences among groups and stocks.

The GSI helps monitor the process of gametogenesis in female teleost fish^43^ and is crucial for assessing how well both the male and female broodstock gonads are developing^44^. Highest GSI in females fed a diet with 10% BSFL meal, whereas the highest GSI in males was in the group receiving 20% BSFL has been reported previously^45^. However, no change in biometry and GSI (except length reduction) in zebrafish with BSF substitution, due to parallel dietary reduction in PUFAs and increase in SFAs^42^. In this study, FM and IM-based diets had similar effects on GSI, but GSI was significantly higher in FR than GS. In males, the highest GSI was observed in GS than in FR, indicating stocks affect GSI, but not diets. Fecundity is a prominent parameter for measuring reproductive capacity. Absolute fecundity was higher in GS than FR, but relative fecundity was highest in FR. The researcher reported that larger *C. magur* females had more eggs but lower relative fecundity^46^, that means variation in fecundity depends on fish size, consistent with our results. The two different feeds (FM and IM-based diets) had similar effects on reproductive indices. Similarly, previous report found that the supplementation of BSFL meal with soybean meal replacement did not affect absolute fecundity, but relative fecundity was higher in a soybean meal-based control diet, which is contradictory to our study^45^.

This study examined reproductive performance parameters including OSI, fertilization rate, hatching rate, and larval survival, across different stocks and treatment groups. These parameters were significantly influenced by stock differences. However, the IM-based diet had an effect comparable to the FM-based diet on reproductive performance. In contrast, egg weight and diameter did not differ significantly across dietary treatments or stocks. Notably, OSI, fertilization rate, hatching rate, and larval survival were significantly higher in FR than in GS. However, genetic selection programs aimed at enhancing growth traits may negatively impact reproductive performance^10^. Similarly, some researchers reported that selection for accelerated growth rates in broiler hens led to negative responses in reproductive traits, especially fertility^22,23^. Furthermore, both IM- and FM-based diets exerted similar effects on reproductive performance in magur broodstock.

Sex steroid hormones are key indicators of gonadal maturity, enhancing reproductive performance^47,48^. Vitellogenesis, regulated by the hypothalamic-pituitary-gonadal axis, involves estradiol promoting vitellogenin synthesis in the liver^49,50^. Vitellogenin is then incorporated into oocytes^51,52^. Estradiol levels rise with vitellogenesis, continuing until early oocyte maturation^32,53^. A sharp decline occurs during post-vitellogenesis, signaling final oocyte maturation and ovulation^48,52,54,55^. This study aligns with these observations, showing maximum estradiol in GS compared to FR, with diets having no effect on estradiol levels. IM-based diet-fed groups in either stock showed effects similar to FM-based diets. This indicates progressive vitellogenesis in GS, with progesterone or 17α20β dihydroxyprogesterone (17α20β-DHP) acting as a maturation-inducing hormone (MIH) in teleosts. 17α20β-DHP induces gamete maturation in fish, triggered by luteinising hormone (LH)^48,56–59^. The rise in 17α20β-DHP determines final gamete maturation^59,60^. In this study, higher levels of 17α20β-DHP were observed in FR, with significantly lower levels in GS. Both diets had the same effect on 17α20β-DHP in both stocks. The higher estradiol and lower 17α20β-DHP in GS indicated delayed maturation compared to FR, with similar effects from both diets. In males, 17α20β-DHP was higher in FR than in GS, with both diets showing the same effect on males. In this study, 11-Keto-testosterone (11-KT) was higher in FR females than in GS; both diets had similar effects on 11-KT. During maturation and ovulation, testosterone levels increase in females^61^. This has been observed in species such as medaka (*Oryzias latipes*)^62^, kutum (*Rutilus frisii*)^63^, carp (*Cyprinus carpio*)^64^, and sturgeon (*Acipenser persicus*)^65^. In this study, GS showed delayed maturation, evident by higher estradiol and lower 17α20β-DHP and 11-KT levels. Conversely, higher 11-KT was observed in GS males compared to FR; both diets gave the same results. Previous report found the highest 11-KT levels in male magur’s fed 10–20% BSFL meals^45^.

Serum glucose levels were higher in FR than in GS, suggesting that FR female magur may have a higher physiological demand or metabolic response. While elevated blood glucose levels are often associated with stress, it is important to note that this increase could also be linked to increased energy expenditure related to reproductive activities^66,67^. Elevated glucose could, therefore, reflect a physiological response to both stress and reproductive energy needs, rather than stress alone^68^. Despite these elevated glucose levels, FR female broodstock exhibited better reproductive performance. Additionally, the IM-based diet had a similar effect on serum glucose levels as the FM-based diet. In contrast, male broodstock did not exhibit significant differences in glucose levels in response to dietary composition or stock variation. These findings align with another reported study^45^, in which no significant changes in serum glucose levels among male broodstocks with different BSFL meal inclusion. TAG, a key lipid subgroup, serves as the primary energy storage form. TAG can be transported to the liver and steroidogenic organs, including ovaries^69^. In this study, serum TAG levels were significantly higher in FR female Magur broodstock than in GS. However, the IM-based diet produced comparable results to the FM-based diet. In contrast, serum TAG levels in male broodstock were unaffected by stock variation and dietary composition. These results were consistent with one researcher, who reported no significant changes in serum TAG levels in male broodstocks with different BSFL meal inclusion^45^. Elevated blood cholesterol levels are associated with reduced gonadal activity in fish^70,71^. Similarly, increased serum cholesterol levels in *Clarias batrachus* during the pre-spawning stage showed the highest cholesterol levels corresponded to the lowest GSI values and vice versa^72^. In the present study, the lowest serum cholesterol levels were observed in both male and female magur broodstock from FR, where the highest GSI values were recorded. Moreover, IM-based diets produced results comparable to FM-based diets.

The *fshr*,* lhr*,* cyp19a1a* and *11β-hsd* genes are important markers of steroidogenesis in the developing and maturing gonads, while *vtg* plays a role in showing vitellogenesis progress. The *11β-hsd* is important for making 11-Keto-testosterone (11-KT) which is the primary androgen in fish^73^. In our study, genes such as cyp19a1a in females, 11β-hsd in males, fshr and lhr in both sexes and vtg were significantly altered among different stocks. In this study, the genes involved in steroidogenesis (*cyp19a1a* in females and *11β-hsd* in males), receptors for sex hormones (*fshr* and *lhr* in both males and females) and *vtg* were significantly different between stocks. Notably, *fshr* and *cyp19a1a* were expressed at much higher levels in female magurs from GS than in those from FR. The process of making estrogen in granulosa cells from testosterone is controlled by the enzyme cytochrome P450 aromatase from the *cyp19a1a* gene and this enzyme is activated by the follicle-stimulating hormone (FSH) signalling pathway^50,74^. FSH binding to its receptor causes genes to be transcribed and aromatase to be synthesised^75^. As a result, FSH controls estrogen production by acting on its receptor (*fshr*) through FSH-stimulated signalling pathways^76^. The parallel expression of *fshr* and *cyp19a1a* coincides with estradiol production in females. This was further supported by the expression of *vtg*, indicating ongoing active vitellogenesis. *vtg* levels often fluctuate with the season in fish and are correlated with oocyte growth^5^. In the case of males, *fshr* and *cyp19a1a* were not affected by different stocks. Conversely, *lhr* expression was much greater in both male and female magur broodstocks from FR than in GS. The development of oocytes is marked by increased plasma LH levels, increased *lhr* expression and an LH-induced change in the ovarian follicle’s steroid production pathway^58^. The expression of *lhr* in FR in our study was found to be linked to the presence of 17α20β-DHP, a hormone that helps maturation. Moreover, levels of *11β-hsd* were significantly higher in GS than in FR. This expression of *11β-hsd* coincides with serum 11KT, indicating that tissues are in the spermatogenic stage. Androgens, such as testosterone and 11-KT, increase gradually as spermatogenesis proceeds and decrease during spermiation^77^. These results clearly show that FR matured much faster than GS; however, IM-based diets affected the expression of *vtg* in females and *11β-hsd* in case of males. However, the IM-based diets were performed at par with the FM-based diets.

Histological analysis of the ovaries and testis confirmed that the different stocks had a significant effect on gonadal maturation. In the FR fed with both FM and IM-based diets, the oocytes were predominantly composed of mature oocytes (MO). The FRFM group showed oocytes characterised by germinal vesicle breakdown (GVBD) stage; similarly, the FRIM group also showed oocytes with germinal vesicle movement (GVM) stage with the nucleolus migrating towards the animal pole. This observation aligns with the increased 17α20β-DHP levels and higher expression of the *lhr* gene. Reduced estradiol levels and the downregulation of *cyp19a1a*, *fshr*, and *vtg* gene expression indicated that the ovaries in the FR groups fed either diet (FM and IM) were mature. In contrast, fish belonging to GS fed either of the diets (FM and IM-based diets) exhibited a higher proportion of vitellogenic oocytes, with the nucleolus situated at the center of the oocyte. This was accompanied by increased serum estradiol levels and decreased 17α20β-DHP levels. These steroidal changes were paralleled by the upregulation of *cyp19a1a* and the downregulation of *lhr* genes, suggesting a shift in endocrine regulation favouring vitellogenesis. Likewise, this finding contradicts the study conducted by one researcher^42^, who reported that substituting fishmeal with up to 50% BSFL meal in female zebrafish resulted in reduced egg production. However, in our study, an IM-based diet was administered at par with FM-based diets. This delay could be due to the downregulation of reproductive traits in the GS.

Histological analysis of the testes revealed significant development and maturation across different stocks. In the FR group, fed diets containing either FM or IM, the lumen of the seminiferous tubules was densely populated with mature spermatozoa (SZ) and a limited number of spermatids (St). These observations correspond with elevated levels of 17α20β-DHP and reduced serum concentrations of 11KT, suggesting a decline in active spermatogenesis, alongside increased maturation and spermiation. This interpretation is further supported by the upregulation of the *lhr* gene in FR fed either FM or IM-based diet. Conversely, in the GS group fed diets containing either FM or IM, the lumen of the seminiferous tubules contained spermatozoa, and spermatids, indicating ongoing active spermatogenesis. According to previous study^78,79^, the progression of spermatogenesis can be delineated by a reduction in cell size and an increase in basophilic staining from spermatogonia to spermatozoa, facilitating the differentiation of developmental stages within the germinal epithelium. Our study is also supported by the histological examinations of testis by one researcher^80^. This finding was corroborated by elevated serum levels of 11KT and reduced 17α20β-DHP concentrations. Downregulation of the *lhr* gene further supported these results.

## Materials and methods

### Ethical approval

All procedures were conducted in compliance with applicable rules and regulations. The utilisation and management of animals adhered to the ARRIVE guidelines. The research followed ethical guidelines and received approval from the Institutional Animal Ethics Committee of ICAR-Central Institute of Fisheries Education, Mumbai-400,061, under approval number 542/CPCSEA.

### Experimental diet, formulation and Preparation

Fish meal (FM) and insect meal (IM) were used as the main protein sources in two nutritionally balanced iso-caloric and iso-nitrogenous practical diets (Table 1). Other protein sources include defatted soybean meal (DSBM), groundnut oil cake (GNOC), and de-oiled rice bran (DORB). Wheat and corn flour served as carbohydrate sources, while a 1:1 mixture of fish and sunflower oils provided lipids. The FM diet comprised 14.65% fish meal, whereas in the IM diet substituted FM protein with 20% BSFL meal. Feed preparation followed the procedure described reported by researcher^13^.

The dough was made by homogenising the ingredients (except for the vitamin-mineral mix and additives) and then steamed. Next, the mixture was mixed with the remaining ingredients (vitamin-mineral mix, oil and additives), made into pellets 3 mm in size, dried at 60 °C and kept at 4 °C until use.

### Experimental setup and design

The experiment was carried out at the Freshwater Fish Farm, Balabhadrapuram, sub-centre of ICAR-Central Institute of Fisheries Education, Kakinada, Andhra Pradesh. Brooders of Magur (*Clarias magur*) aged 1 + years, from genetically selected (GS) and farm-raised (FR) stocks (non-selected) were obtained from the same farm, where farm-bred populations were maintained under controlled conditions. The brooders were subjected to a 24-hr fasting period prior to the feeding trial. FR and GS groups, with similar body weights (131.19 to 134.33 g and 198.33 to 200.60 g, respectively), were randomly allocated to four groups viz. FRFM, FRIM, GSFM, and GSIM, with three replicates each (12 cemented tanks, 1 m × 4 m × 1.5 m). Each tank housed 14 fish with a stocking density of 3 fish/m² (7 females and 7 males), following a 2 × 2 factorial experimental design, with three replicates for each treatment. Brooders were fed to satiation, with the amount of feed adjusted daily to ensure that each fish could consume as much as needed. This feeding regime was chosen to mimic natural conditions and provide the broodstock with optimal nutrition. Feed intake was closely monitored and adjusted to minimize significant deviations within each treatment group. Each treatment group included three replicates to account for individual variation in feed consumption, and statistical analysis was performed considering these variations. Twice a day, at 07:00 and 18:00 h, fish were fed manually to satiation, and their feed consumption was noted. The experimental period continued for 120 days. Water parameters were maintained at optimum as water temperature (25–28 °C), pH (7.5–8.5) and dissolved oxygen (6.1–7.5 mg/L) throughout the experimental period.

### Sample collection and tissue processing

After the study ended, the fish were starved for 24 h. Each fish in all the tanks was weighed and counted. The chosen fish were measured for weight and length to find their condition factor. Two fish were selected euthanized using clove oil (concentration = 2 ppm/5 L) according to the previously reported protocol^10^. The relevant indices were calculated using morphometric parameters and weights of the liver, visceral fat and gonads. The gonadal tissue (100 mg) was preserved into RNAlater^®^ and stored at − 80 °C for molecular studies. A blood sample were collected for serum analysis and parts of the gonads were put in 10% neutral buffered formalin (NBF) for histology. The rest of the gonadal tissue was preserved at −20 °C for analysis of its fatty acids.

### Proximate and fatty acid composition of feed

The proximate composition of diets was analyzed as per AOAC procedures^81^. Digestible energy was calculated using the method described by Halver^82^. FAME samples of diets and ovary samples for quantification of fatty acids were outsourced to NABL accredited laboratory ENVIROCARE, Thane, Maharashtra.

### Growth and nutrient utilization

To evaluate growth, the initial weight of magur broodstock was measured at stocking and again every 15 days. An electronic balance was used to record weight and indices of growth and nutrient utilization were calculated by using these formulas:

$$\:\text{Weight}\:\text{gain}\:\%\:=\:\text{Final}\:\text{wet}\:\text{weight}\:\left(\text{g}\right)\text{-Initial}\:\text{wet}\:\text{weight}\:(\text{g})\:$$

$$\:\text{Weight}\:\text{gain}\:\%=\frac{\text{Final}\:\text{wet}\:\text{weight}\:\left(\text{g}\right)\text{-Initial}\:\text{wet}\:\text{weight}\:(\text{g})\:}{\text{Ininital}\:\text{wet}\:\text{weight}\:(\text{g})}{\times \:100}$$

$$\:\text{Specific}\:\text{growth}\:\text{rate}\:(\text{SGR},\%)=\frac{\text{(ln}\:\text{of}\:\text{final}\:\text{weight-ln}\:\text{of}\:\text{initial}\:\text{weight)}}{\text{duration}\:\text{of}\:\text{the}\:\text{experiment}\:(\text{days})}{\times \:100}$$

$$\:\text{Feed}\:\text{conversion}\:\text{ratio}\:(\text{FCR},\:\%)\:=\frac{\text{Feed}\:\text{consumption}\:(\text{g}\:\text{on}\:\text{dry}\:\text{matter}\:\text{basis})\:}{\text{Body}\:\text{weight}\:\text{gain}\:(\text{g}\:\text{on}\:\text{wet}\:\text{weight}\:\text{basis})}$$

$$\:\text{Protein}\:\text{efficiency}\:\text{ratio}\:(\text{PER})\:=\frac{\text{Wet}\:\text{weight}\:\text{gain}\:(\text{g})\:}{\text{Protein}\:\text{consumption}\:(\text{g}\:\text{on}\:\text{dry}\:\text{matter}\:\text{basis})}{\times100}$$

$$\:\text{Condition}\:\text{Factor}\:(\text{K})\:=\frac{\text{Final}\:\text{wet}\:\text{weight}\:\left(\text{g}\right)\text{}}{{\text{Length}\:(\text{cm})\:}^{3}}{\times100}$$

### Reproductive indices

After sample collection, reproductive indices such as the hepatosomatic index (HSI), gonadosomatic index (GSI), relative fecundity and absolute fecundity were calculated using standard formulas. Oocyte diameter was measured using graduated slides under a microscope (Nikon, V12BDC, Japan), and the average oocyte weight was determined from pooled samples.

$$\:\text{Gonadosomatic}\:\text{index}\:(\text{GSI},\:\%)\:=\:\text{Gonad}\:\text{Weight}\:(\text{wet}\:\text{weight})/\text{Fish}\:\text{weight}\:(\text{wet}\:\text{weight})\:\times\:100\:$$

$$\:\text{Hepatosomatic}\:\text{index}\:(\text{HSI},\:\%)\:=\:\text{Liver}\:\text{weight}\:(\text{wet}\:\text{weight})/\:\text{fish}\:\text{weight}\:(\text{wet}\:\text{weight})\:\times\:100\:$$

$$\:\text{Absolute}\:\text{fecundity}\:(\text{Total}\:\text{eggs})\:=\:\text{Total}\:\text{number}\:\text{of}\:\text{eggs}\:\text{in}\:1\:\text{gram}\:\text{egg}\:\text{sample}\:\times\:\text{Ovary}\:\text{wet}\:\text{weight}$$

$$\:\text{Relative}\:\text{fecundity}\:(\text{eggs}\:/\:100\:\text{g}\:\text{of}\:\text{fish})=\frac{\text{Absolute}\:\text{fecundity}\:}{\text{Female}\:\text{body}\:\text{weight}\:(\text{g})}\times\text{100}$$

### Artificial spawning and insemination procedures

The brooders for induced spawning were selected based on their maturity, which was assessed through physical characteristics (size and gonadal development). Only brooders exhibiting signs of maturity were chosen for the induced spawning procedure to ensure reliable reproductive outcomes. Following the experiment, induced breeding was carried out using OVATIDE^®^ (sGnRH analogue Hemmo Pharmaceuticals Pvt. Ltd, India) following previously reported procedure^10^. Two pairs per tank (2 males and 2 females) were selected. Females received 0.2 mL per 100 g body weight and males received half the dose intramuscularly. Males and females were housed together in fiberglass-reinforced plastic (FRP) tanks until gamete collection. The eggs were stripped after 17 h in dry plastic trays. As males did not release milt naturally, the testes were surgically excised after anesthetizing the fish in 5 L of water containing 2 ppm clove oil. The sperm suspension was prepared by macerating the testes in 0.9% NaCl according to previous report^83^. Gametes were mixed using feathers and activated with water (100 mL), and the fertilized eggs were rinsed with fresh water to remove any residual milt and then transferred to incubation trays. During egg incubation, water temperature was maintained at 27–30 °C, and dissolved oxygen (DO) levels were kept between 6 and 7 mg/L. pH levels were also monitored and maintained (7.5–8.5) between optimum range^84^. These parameters were checked regularly to ensure optimal conditions for egg development and hatching.

### Reproductive performance

Eggs were identified as fertilized at 12–15 h after fertilization, with transparent eggs indicating fertilization while opaque eggs showing no fertilization. To count the eggs and measure the fertilization rate, we examined the eggs by direct visual enumeration. In every incubation tray, approximately 300 eggs were put and examined with a magnifying lens. The number of fertilized eggs was counted, and the fertilization rate was determined with a standard calculation. The hatchlings were visually counted and a few samples were siphoned and put into a white enamel bowl to make the process more accurate. Subsequently, the hatching rate was calculated by comparing the number of hatchlings to the number of eggs laid. On the third day post-hatch (3 dph) and again on the seventh day post-hatch (7 dph), larval survival was assessed to see how maternal nutrition influence the results. To ensure all the data was interpreted the same way, survival rates were calculated using a standard formula:

$$\:\text{Ovi}\text{-somatic}\:\text{index}\:\left({\%}\right)\text{=}\frac{\text{Striped}\:\text{weight}\:\text{of}\:\text{eggs}\:\left(\text{g}\right)}{\text{Body}\:\text{weight}\:\text{of}\:\text{the}\:\text{female}\:\left(\text{g}\right)}\times\:\text{100}$$

$$\:\text{Fertilization}\:\text{rate}\:(\%)\:=\frac{\text{Number}\:\text{of}\:\text{fertilized}\:\text{eggs}}{\text{Total}\:\text{number}\:\text{of}\:\text{eggs}\:(\text{fertilized}\:+\:\text{unfertilized})\:}\times\text{100}$$

$$\:\text{Hatching}\:\text{rate}\:(\%)\:=\frac{\text{Number}\:\text{of}\:\text{hatchlings}}{\text{Total}\:\text{number}\:\text{of}\:\text{fertilized}\:\text{eggs}\:}\times\text{100}$$

$$\:\text{Larval}\:\text{survival}\:\text{rate}\:(\%)\:\frac{\text{Number}\:\text{of}\:\text{survived}\:\text{larvae}}{\text{Total}\:\text{Number}\:\text{of}\:\text{larvae}\:\text{stocked}\:}\times\text{100}$$

### Determination of serum sex steroid hormones

The sex steroid hormones 17-β estradiol (E2), 11-Keto-testosterone (11-KT) and 17α,20β-dihydroxyprogesterone were measured using an enzyme-linked immunosorbent assay (ELISA) kit from Krishgen Biosystems (Mumbai, Maharashtra, India). All serum samples were allowed to reach room temperature and then gently mixed by inverting them to ensure sample homogeneity before analysis. All assays were done exactly as the manufacturer recommended protocol. An 800 TS microplate reader (BioTek Instruments, USA) was used to measure optical density (OD), and the concentrations of the hormone were calculated by using standard calibration curves with known concentrations.

### Serum biochemical parameters

Serum total triglyceride (TAG) concentrations were determined using the Erba Triglyceride Assay Kit (ERBA Diagnostics, Mannheim, Germany), following the previously reported methodology^85^. In brief, 5 µL of the serum sample was mixed with 500 µL of assay reagent and incubated at 37 °C for 10 min. A reagent blank (distilled water) and standard were processed under identical conditions. Absorbance was measured at 546 nm using the blank as a reference. TAG concentrations were expressed in mg/dL and calculated using a standard calibration curve based on the following formula:

$$\:\text{Triglycerides}\:(\text{mg/dl})\:=\:\frac{\text{Absorbance}\:\text{of}\:\text{test}\:(\text{T})}{\text{Absorbance}\:\text{of}\:\text{standard}\:(\text{s})}\:\times\:\text{Concentration}\:\text{of}\:\text{standard}(\text{mg/dl)}$$

Serum total cholesterol levels were estimated using the Liquixx Cholesterol Kit (BLT00034, Erba Mannheim, Transasia BioMedicals Pvt. Ltd., Himachal Pradesh, India), employing the enzymatic-colorimetric method reported previously^86^. The absorbance of both test samples and standards was measured at 505 nm, using the reagent blank as a reference. Cholesterol concentrations were calculated using the following equation and expressed in mg/dL:

$$\:\text{Cholesterol}\:(\text{mg/dl})\:=\:\frac{\text{Absorbance}\:\text{of}\:\text{test}\:(\text{T})}{\text{Absorbance}\:\text{of}\:\text{standard}\:(\text{s})\:}\times\:\text{Concentration}\:\text{of}\:\text{standard}$$

Serum glucose concentrations were measured using the Liquixx Glucose Kit (Erba Diagnostics Mannheim, Transasia BioMedicals Ltd., Solan, Himachal Pradesh, India), following the reported method^87^. Glucose levels were expressed in g/dL, calculated using the formula:

$$\:\text{Glucose}\:(\text{g/dl})\:=\frac{\text{Absorbance}\:\text{of}\:\text{test}\:\left(\text{T}\right)}{\text{Absorbance}\:\text{of}\:\text{standard}\:(\text{s})\:}\:\:\times\:\text{Concentration}\:\text{of}\:\text{standard}(\text{100mg/dl})$$

### Gene expression analysis

Reproductive genes *fshr*,* cyp19a1a*,* vtg*,* lhr* and *11β-hsd* were examined for expression in both female and male ovarian and testicular tissues respectively in all experimental groups. The primers used for the quantitative RT-PCR (qRT-PCR) were taken from the previous study which were selected based on their successful application in similar studies^13^. We have also included the primers in the supplementary file. First, the gene-specific primers were reconstituted in sterile Tris-EDTA (TE) buffer (pH 7.0) at 100 pmol/µL and then diluted with nuclease-free water to 10 pmol/µL for working concentration. Total RNA was extracted from gonadal tissues by using TRIzol™ reagent (Invitrogen, USA) with a modified protocol from another study^88^. NanoDrop was used to measure the quantity and purity of the isolated RNA, with concentrations expressed as ng/µL and purity as A260/A280 absorbance ratio. The integrity of RNA was confirmed by running it on a 1% agarose gel using electrophoresis. Genomic DNA contamination was prevented by treating the RNA samples with RNase-free DNase I (Thermo Scientific, USA) before making complementary DNA (cDNA). The protocol given with the ExcelRT ™ Reverse Transcription Kit II (SMOBIO, Taiwan) was followed to perform first-strand cDNA synthesis using 1 µg of total RNA. The quantitative real-time PCR (qRT-PCR) was done in a 10 µL reaction containing 0.5 µL of each of forward and reverse primers (5 pmol), 3 µL of nuclease-free water, 5 µL of cDNA and L TB Green™ Premix Ex Taq (Tli RNaseH Plus). Amplification was performed using the AriaMx Real-Time PCR System (Agilent Technologies, USA). The thermal cycling program was: 95 °C for 10 s for denaturation, then 40 amplification cycles involving of denaturation at 95 °C for 15 s, annealing at the primer-specific melting temperature (Tm) and extension at 72 °C for 2 min. Relative expression of the genes was calculated using the 2^−ΔΔCT method^89^, with β-actin (*actb*) as the reference gene.

$$\:\Delta\text{CT}\:=\:\text{target}\:\text{gene}\:\text{CT}\:\text{value}\:-\:\text{reference}\:\text{gene}\:\text{CT}\:\text{value}$$

$$\:\Delta\Delta\text{CT}\:\text{value}\:=\:\Delta \text{CT}\:\text{value}\:\text{of}\:\text{treatment}\:\text{group}\:-\:\Delta\text{CT}\:\text{value}\:\text{of}\:\text{control}\:\text{group*}$$

*The farm-raised stock fed FM-based diet (FRFM) group was used as a control in our study.

### Histological examination of gonadal tissues

Histological analysis of gonadal tissues was carried out across all experimental groups. Gonads from both male and female fish were collected following standard dissection procedures and immediately fixed in 10% neutral buffered formalin (NBF) for a period of 24 h. After the initial fixation, the solution was replaced with fresh NBF, followed by a second replacement one week later. The samples were preserved in NBF for up to one month, with routine monthly replacements to ensure tissue integrity prior to further processing. Fixed gonadal tissues were submitted to the Mumbai Histology Centre for routine histological processing. Histological analysis was performed on gonadal tissues, which were fixed in 10% neutral buffered formalin, embedded in paraffin, and sectioned at a thickness of 5 μm. The sections were stained with Hematoxylin and Eosin (H&E) for evaluation^80^. Processed tissue sections were examined microscopically using an Olympus FSX100 imaging system to evaluate gonadal structure and cellular morphology.

### Statistical analysis

The data were subjected to analyze by One-way and Two-way analysis of variance (ANOVA) using a software SPSS 22.0 for Windows. Duncan’s multiple range test under post-hoc was used for observing significant differences among the mean values at 5% probability level (*p* < *0.05*). The analysed data was expressed as Mean and SEM (Two-way), and mean ± SE (One-way).

Table 2 presents data where only a single factor was of interest, and hence, a One-Way ANOVA was used to analyze the differences between groups in individual parameters. This approach allows for evaluating the effects of a single independent variable on the outcome measures. On the other hand, Tables 3, 4, 5, 6, 7 and 8 include datasets involving two independent variables, for which both One-Way and Two-Way ANOVA were employed. The One-Way ANOVA was used to analyze the main effects of each independent variable, while the Two-Way ANOVA enabled the examination of both the main effects of the two factors (different stocks and different feeds) and their interaction. This dual approach provided a comprehensive analysis of how multiple variables or conditions impacted the outcome measures.

## Conclusion

The GS showed significantly better growth performance, including higher final body weight, weight gain, and feed efficiency, regardless of the diet. However, the FR outperforms GS in terms of higher reproductive performance, including higher gonadosomatic index (GSI), relative fecundity, larval survival, fertilisation and hatching rate as well as expression of reproduction-related genes compared to the GS stock irrespective of diets. In terms of diet, the replacement of fishmeal with IM did not significantly affect the reproductive performance or growth of either stock. Therefore, insect meal can be considered a viable alternative to fishmeal in magur brooder diets without compromising growth or reproductive output. These findings suggest that while selecting fish on the basis of growth traits, reproductive traits also to be considered to ensure successful breeding and seed production.

## Supplementary Information

Below is the link to the electronic supplementary material.

Supplementary Material 1

## Data Availability

The data that support the findings of this study are available from the corresponding author upon reasonable request.
