# Supplementary material for: Black soldier fly larvae meal influences the growth, reproduction and related gene expression in farm-raised and growth-trait-selected Clarias magur brooders
Source: Sci Rep. 2025 Dec 20;16:775. doi: 10.1038/s41598-025-30296-8 (PMC12780206; doi:10.1038/s41598-025-30296-8)
Supplement: Supplementary file 1 — Supplementary Material 1 [file 41598_2025_30296_MOESM1_ESM.docx]

**Supplementary table: Primers of *fshr*, *cyp19a1a*, *lhr*, *vtg*, and *11β-HSD* genes used for real-time PCR analysis in female and male *Clarias magur***

| **Primer** | **Sequence** | **Accession no** | **Amplicon size (bp)** |
| --- | --- | --- | --- |
| ***actb* Forward** | GAAGGTTATGCCCTGCCCCATGCC | EU52790.2 | 122 |
| ***actb* Reverse** | TCCCTCTCGGCTGTGGTGGTGAAG |  |  |
| ***fshr* Forward** | CATCCGCAACCAGGAGCCATAC | MF373415.1 | 138 |
| ***fshr* Reverse** | CACCAGATGAGGACACGCAGGA |  |  |
| ***cyp19a1a* Forward** | CCCAAACCCTCAGAGTTCAGC | U687002.1 | 100 |
| ***cyp19a1a* Reverse** | TACCAACACAGGAACGAGGACC |  |  |
| ***lhr* Forward** | GATATTGCTGGGTTTGGGTTTC | MF373413.1 | 140 |
| ***lhr* Reverse** | AGATGACACATGAGGAAGCG |  |  |
| ***vtg* Forward** | CAAAGACCTGAACAACTGCCA | KJ845350.1 | 145 |
| ***vtg* Reverse** | ACCTTTGTCAGTGGGCTTCAT |  |  |
| ***11β-hsd* Forward** | AGTACCTGCTCTCCTCGCCTGACCTTG | XM_053942487.1 | 110 |
| ***11β-hsd* Reverse** | CGTCTCCAAAGTTCACACACACTCCTGC |  |  |

*actb*, β-actin; *fshr*, Follicle stimulating hormone receptor; *cyp19a1a*, Cytochrome p450 aromatase; *lhr*, Luteinising hormone receptor; *vtg*, Vitellogenin; *11β-HSD*, 11β Hydroxysteroid dehydrogenase
